# The differential expression of micro-RNAs 21, 200c, 204, 205, and 211 in benign, dysplastic and malignant melanocytic lesions and critical evaluation of their role as diagnostic biomarkers

**DOI:** 10.1007/s00428-020-02817-5

**Published:** 2020-05-09

**Authors:** Katherine Quiohilag, Peter Caie, Anca Oniscu, Thomas Brenn, David Harrison

**Affiliations:** 1 Department of Pathology, Royal Infirmary of Edinburgh, 51 Little France Crescent, Edinburgh, EH16 4SA UK; 2 School of Medicine, University of St Andrews, St Andrews, UK; 3 Departments of Pathology & Laboratory Medicine and Medicine and The Arnie Charbonneau Cancer Institute, Cumming School of Medicine, University of Calgary, Calgary, Canada

**Keywords:** microRNA, Molecular pathology, Melanoma, Dermatopathology

## Abstract

Overlapping histological features between benign and malignant lesions and a lack of firm diagnostic criteria for malignancy result in high rates of inter-observer variation in the diagnosis of melanocytic lesions. We aimed to investigate the differential expression of five miRNAs (21, 200c, 204, 205, and 211) in benign naevi (*n* = 42), dysplastic naevi (*n* = 41), melanoma in situ (*n* = 42), and melanoma (*n* = 42) and evaluate their potential as diagnostic biomarkers of melanocytic lesions. Real-time PCR showed differential miRNA expression profiles between benign naevi; dysplastic naevi and melanoma in situ; and invasive melanoma. We applied a random forest machine learning algorithm to classify cases based on their miRNA expression profiles, which resulted in a ROC curve analysis of 0.99 for malignant melanoma and greater than 0.9 for all other groups. This indicates an overall very high accuracy of our panel of miRNAs as a diagnostic biomarker of benign, dysplastic, and malignant melanocytic lesions. However, the impact of variable lesion percentage and spatial expression patterns of miRNAs on these real-time PCR results was also considered. In situ hybridisation confirmed the expression of miRNA 21 and 211 in melanocytes, while demonstrating expression of miRNA 205 only in keratinocytes, thus calling into question its value as a biomarker of melanocytic lesions. In conclusion, we have validated some miRNAs, including miRNA 21 and 211, as potential diagnostic biomarkers of benign, dysplastic, and malignant melanocytic lesions. However, we also highlight the crucial importance of considering tissue morphology and spatial expression patterns when using molecular techniques for the discovery and validation of new biomarkers.

**Electronic supplementary material:**

The online version of this article (10.1007/s00428-020-02817-5) contains supplementary material, which is available to authorized users.

## Introduction

Malignant melanoma has seen a rapid increase in incidence in the last several decades, having risen by almost 50% in the last decade alone, and is now the fifth most common cancer in the UK [1]. The current gold standard method for the diagnosis of melanocytic lesions is histological examination of tissue by pathologists. However, this has been described as ‘one of the most challenging and controversial fields in diagnostic histopathology’ [2] due to overlapping histological features between benign and malignant lesions [3] and lack of firm diagnostic criteria for malignancy. This creates the potential for both the over- and underdiagnosis of malignancy.

Dysplastic naevi represent a particular challenge. Histologically, they have overlapping features with both benign naevi at the mild end of the spectrum and melanoma at the severe end. Accordingly, various studies have shown high rates of interobserver variation in their histological diagnosis [4, 5]. Of particular interest is a recent major study of 187 pathologists which concluded, in agreement with previous smaller studies, that diagnosis of dysplastic naevi and early-stage invasive melanoma was neither reproducible nor accurate [6]. Therefore, a reliable and objective molecular marker to aid pathologists’ visual assessment of benign naevi, dysplastic naevi, and melanoma would be of great clinical value.

MicroRNAs (miRNAs) have been described as possessing ‘most of the characteristics of an ideal biomarker’: they are sensitive and specific [7] and are stable in FFPE (formalin-fixed paraffin-embedded) tissue [8–11]. Over the last decade, the role of miRNAs as potential biomarkers in melanoma has attracted significant attention, with miRNAs found to have potential as diagnostic, prognostic, and predictive biomarkers, as well as putative therapeutic targets [9, 10, 12–15].

miRNAs are short (up to 22 nucleotides), non-coding RNA molecules present in all cells. They function in post-transcriptional regulation of gene expression by binding to the 3′ untranslated region (UTR) of mRNAs, blocking translation and sometimes accelerating mRNA degradation. Depending on their target, miRNAs can function as both tumour suppressors and tumour promoters. They provide a complex and powerful means of regulating gene expression, with computational predictions suggesting that they regulate one-third of the genome [16].

Although differential expression of various miRNAs has been demonstrated in benign and malignant melanocytic lesions, there is limited description of miRNA expression in dysplastic naevi and one of their major differential diagnoses, melanoma in situ [17–20], and studies which have been published tend to suffer from small cohort sizes. Given the significant degree of diagnostic uncertainly generated by these lesions, we aimed to investigate the role of a panel of five miRNAs as a potential diagnostic biomarker of benign naevi, dysplastic naevi, melanoma in situ, and invasive melanoma.

## Methods

### Ethical approval and cohort selection

Ethical approval for access to FFPE (formalin-fixed paraffin-embedded) archival tissue was gained from the NRS BioResource and Tissue Governance Unit (SR961), and permission to conduct the study was gained from ACCORD via the Integrated Research Application System (IRAS). All cases were identified by searching the NHS Lothian Pathology archive. All diagnoses were confirmed by dermatopathologist review. Cases with insufficient lesional tissue for macrodissection and molecular analysis, defined in this study as lesions with a tumour percentage below 10%, were excluded. The final cohort consisted of 42 cases of melanoma; 42 cases of melanoma in situ; 41 cases of dysplastic naevi; 42 cases of benign common naevi; and 17 cases of normal skin.

All morphological subtypes of melanoma with a vertical growth phase were included and detailed clinicopathological features were recorded (summarised in Table 1, with full details in ESM 1). Similarly, different morphological subtypes of melanoma in situ were included, with the final cohort consisting of 28 cases of melanoma in situ and 14 cases of lentigo maligna. Because of the known high level of diagnostic discordance of dysplastic naevi and the resulting potential for inconsistent use of this term between pathologists, inclusion criteria for this group were strict during dermatopathology review and were based on the WHO classification of skin tumours. All included cases had evidence of cytological atypia and displayed classical architectural features of dysplastic naevi including subepidermal fibroplasia; bridging of adjacent rete ridges; lentiginous proliferation of melanocytes; and shouldering in compound lesions. The final cohort consisted of 13 cases with mild atypia, 13 with moderate atypia, and 15 with severe atypia. Benign common naevi were intradermal or compound naevi without features of congenital type neavus.

**Table 1:** Summary of clinicopathological features of melanoma cohort. *LVI*, lymphovascular invasion

Morphological subtype	Median Breslow thickness	Mean mitotic count (per mm^2^)	Number with ulceration	Number with regression	Number with precursor naevus	Number with LVI	Number with micro-satellites	Immune response
18 nodular	3.6	7	18	5 full	3	3	0	2 brisk
18 superficial spreading				1 partial				15 non-brisk
2 desmoplastic 2 acral lentiginous								25 absent
1 lentigo maligna melanoma								
1 desmoplastic and spindle cell								

All eligible cases were reviewed by a molecular pathologist and lesional areas for macrodissection were identified.

### Nucleic acid extraction

Following microdissection, RNA was extracted using the Recoverall Total Nucleic Acid Isolation Kit for FFPE (AM1975, Thermo Fisher Scientific, UK) according to the manufacturer’s protocol, with the exclusion of deparaffinisation steps. In summary, samples were digested in protease for 15 min at 50 °C (increased to 45 min for small lesions) followed by 15 min at 80 °C. Samples were incubated with DNase mix for 30 min at room temperature prior to further washing and elution. The concentration of RNA extract was measured using a Qubit Fluorometer with the high sensitivity RNA assay.

### Reverse transcription and real-time PCR

miRNA expression levels were assessed using the TaqMan® small RNA assay protocol from Applied Biosystems. Assays specific to miR-21 (hsa-miR-21, assay ID 00397); miR-200c (hsa-miR-200c, assay ID 002300); miR-204 (hsa-miR-204, assay ID 000508); miR-205 (hsa-miR-205, assay ID 000509); and miR-211 (hsa-miR-211, assay ID 000514) were used, with U6 (RNU6B, assay ID 001093) as an endogenous control (Thermo Fisher Scientific). Validation experiments were performed for each assay with serial tenfold dilutions of one sample, run in triplicate, to ensure the amplification efficiency of the endogenous control (U6) and each target miRNA was approximately equal. For all miRNA assays, the plot of ΔCT against log input resulted in a semi-log regression line with a slope < 0.1.

Reverse transcription was carried out using the TaqMan™ MicroRNA Reverse Transcription Kit (catalogue number 4366596, Thermo Fisher Scientific). All RNA samples were diluted in nuclease-free water to 1 ng in 5 μl, with each 15 μl of reverse transcription reaction consisting of 5 μl of RNA, 7 μl of reverse transcription mastermix, and 3 μl of reverse transcription primer.

Real-time PCR was carried out on 96-well plates using TaqMan® Universal PCR Master Mix II, No UNG (catalogue number 4440043, Thermo Fisher Scientific) with each 20-μl PCR reaction made up as follows: 1-μl TaqMan small RNA assay; 1.33-μl product from reverse transcription reaction; 10-μl PCR mastermix; and 7.67-μl nuclease-free water. Samples were run on an Applied Biosystems™ 7500 SDS real-time PCR machine (Thermo Fisher Scientific). All reactions were carried out in duplicate with negative controls.

### In situ hybridisation

Chromogenic in situ hybridisation was carried out on a subset of cases (benign naevi (*n* = 6), dysplastic naevi (*n* = 6; 3 with mild atypia, 1 with moderate atypia, 2 with severe atypia), melanoma in situ (*n* = 6), and melanoma (*n* = 6)) using BaseScope™ assays (ACDbio) specific to each miRNA of interest according to the manufacturer’s protocol. PPIB probes were used as positive controls and DAPB as negative controls (ESM 2 and 3).

In summary, for pre-treatment, slides were incubated with 5–8 drops of hydrogen peroxide at room temperature for 10 min and bathed in antigen retrieval solution within the Braun Multiquick FS 20 steamer for 15 min. Following air drying, a hydropic barrier was drawn around the tissue edge. The next day, the slides were incubated with protease IV at 40 °C for 45 min, with re-application of the protease after 30 min, followed by incubation with the relevant probe at 40 °C for 2 h. The probes were amplified by the addition of AMPS 0–6 in turn as follows: AMP 0 for 30 min at 40 °C; AMP 1 for 15 min at 40 °C; AMP 2 for 30 min at 40 °C; AMP 3 for 30 min at 40 °C; AMP 4 for 15 min at 40 °C; AMP 5 for 45 min at room temperature; and AMP 6 for 15 min at room temperature. Slides were then incubated with RNAScope fast RED solution for 10 min at room temperature and counterstained with haematoxylin and lithium carbonate. Following drying overnight, the spatial distribution of transcripts was assessed by two independent pathologists.

### Statistical analysis

The comparative CT method was applied to calculate ΔΔCT and expression fold changes in each miRNA. If the fold change was between 0 and 1, indicating negative fold change in expression, the formula − 1/2-ΔΔCT was used for linear transformation. Statistical analysis of real-time PCR results was performed using MiniTab version 17. Relative expression of each miRNA in each sample was calculated using the formula 2-ΔCT, followed by log transformation (base 2). Normality was confirmed with the Anderson-Darling tests and probability plots. Welch’s ANOVA followed by the Games-Howell pairwise comparison (with 95% confidence intervals) was used to compare the expression levels of each miRNA between groups. Adjusted *p* values are provided for pairwise comparisons. Tests of equal variance followed by 1-way ANOVA and Tukey’s pairwise comparison were used to compare the expression levels of each miRNA in dysplastic naevi with mild, moderate, and severe atypia. A two-tailed *t* test was used to compare expression patterns in different morphological subtypes of melanoma and melanoma in situ. Correction for lesion percentage was performed by dividing relative expression by the lesion percentage prior to log transformation.

### Decision tree modelling

Random forest with 100 trees was performed with log-transformed relative expression data using Weka open-source machine learning software version 3.6.13. Tenfold cross-validation was used to avoid overfitting. Receiver operating characteristic (ROC) curve analysis was used to evaluate overall accuracy as a diagnostic test.

## Results

### Expression of miRNAs in benign naevi, dysplastic naevi, melanoma in situ, and melanoma

**Fig. 1:**
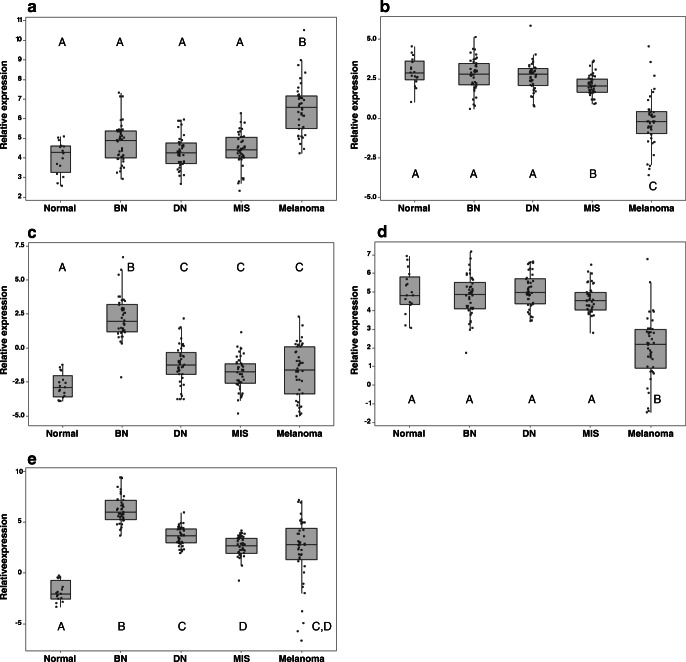
Boxplots of log-transformed relative expression of microRNAs measured with real-time PCR. miRNAs 21 (**a**); 200c (**b**); 204 (**c**); 205 (**d**); and 211 (**e**) in normal skin, benign naevi (BN), dysplastic naevi (DN), melanoma in situ (MIS), and melanoma. Letters indicate outcome of Welch’s ANOVA followed by Games-Howell pairwise comparisons. Groups which do not share a letter are significantly different from each other (*p* < 0.05)

The expression of miRNA 21 was significantly increased in melanoma compared with that in all other groups (*p* < 0.001). Conversely, there was a significant reduction in the expression of both miRNA 200c and 205 in melanoma compared with that in all other groups (*p* < 0.001). The expression of miRNA 204 and 211 was significantly higher in benign naevi compared with that in all other groups (*p* < 0.001) (Fig. 1). The expression of miRNA 200c also showed significantly lower expression in melanoma in situ compared with that in dysplastic naevi (*p* < 0.05) and benign naevi (*p* < 0.05). For miRNA 211, expression was significantly reduced in melanoma in situ compared with that in dysplastic naevi (*p* < 0.001), though owing to a wide variation in expression in the melanoma cohort, neither of these was significantly different from melanoma. There were no significant differences in the expression of miRNA 21 and 205 in benign naevi, dysplastic naevi, and melanoma in situ, and no significant differences in the expression of miRNA 204 in dysplastic naevi, melanoma in situ, and melanoma (Fig. 1).

Examination of miRNA expression in the form of fold changes reveals a 3.41-fold increase in miRNA 21 in melanoma compared with that in benign naevi, with almost 8- and 7-fold reduced expression of miRNA 200c and 205, respectively (Table 2). The greatest fold changes are seen in miRNA 204 and 211, with almost 15- and 16-fold reduced expression respectively in melanoma compared with that in benign naevi. Large negative fold changes in the expression of miRNAs-204 and 211 are also seen in dysplastic naevi and melanoma in situ compared with those in benign naevi.

**Table 2:** Fold changes in expression of each micro-RNA in dysplastic naevi, melanoma in situ, and melanoma compared with benign naevi

	mRNA 21	miRNA 200c	miRNA 204	miRNA 205	miRNA 211
Dysplastic naevi	− 1.39	− 1.07	− 11.04	1.20	− 6.33
Melanoma in situ	− 1.30	− 1.58	− 16.99	− 1.12	− 12.56
Melanoma	3.41	− 7.57	− 14.96	− 6.66	− 15.96

Using 1-way ANOVA with Tukey’s pairwise comparisons, there was no significant difference between the expression levels of all five miRNAs in dysplastic naevi with mild, moderate and severe atypia. Two-tailed *t* tests showed no significant differences in the expression of any miRNAs between different morphological subtypes of malignant melanoma or melanoma in situ.

Overall, dysplastic naevi and melanoma in situ showed a similar pattern of expression, generally grouping with benign naevi for miRNAs 21, 200c, and 205, and with melanoma for miRNAs 204 and 211.

### Expression of miRNAs in normal skin

miRNAs 21, 200c, and 205 showed a similar pattern of expression to that seen in benign and dysplastic naevi and were significantly different from melanoma (Fig. 1; *p* < 0.001). miRNA 200c expression in normal skin was also significantly higher than that of melanoma in situ (*p* < 0.05). miRNA 204 and 211, however, showed significantly lower levels of expression in normal skin than they did in benign naevi and dysplastic naevi (*p* < 0.001). miRNA 211 expression in normal skin was also significantly lower than that in the melanoma in situ and melanoma cohorts (*p* < 0.001), as was miRNA 204 expression but to a slightly lesser extent (*p* < 0.05).

### Decision tree modelling

Random forest was able to correctly classify 36 out of 42 melanomas following tenfold cross-validation (Table 3). Four of the six false-negative melanomas were classified as melanoma in situ, resulting in only two cases which were incorrectly classified as benign. There was one false-positive melanoma which was a melanoma in situ, meaning no benign lesions were incorrectly classified as malignant. Random forest gave very high ROC areas of 0.99 for melanoma, 0.97 for benign naevi, 0.90 for dysplastic naevi, and 0.91 for melanoma in situ, indicating overall high accuracy of this panel of miRNAs as a diagnostic test.

**Table 3:** Confusion matrix from random forest with tenfold cross-validation**.** Each horizontal row shows how cases from each group (*BN*, benign naevi; *DN*, dysplastic naevi; *MIS*, melanoma in situ) were classified based on the miRNA expression data

	Classified as normal	Classified as BN	Classified as DN	Classified as MIS	Classified as melanoma
Normal	16	0	1	0	0
BN	0	39	2	1	0
DN	0	4	27	10	0
MIS	1	1	11	28	1
Melanoma	0	2	0	4	36

### The impact of lesion percentage

One limitation of molecular testing with real-time PCR is that, despite macrodissection, cell types other than melanocytes will inevitably be incorporated into the samples. This effect will be greater in cases with a low lesion percentage. After macrodissection, the average lesion percentage for the dysplastic naevus cohort was 30% and 20.5% for the melanoma in situ cohort. This compared to 65.6% for the melanoma cohort and 62.5% for the benign naevus cohort. Despite the likely impact of lesion percentage on the measured levels of the miRNAs with real-time PCR, no adjustment for this was identified in the published literature.

**Fig. 2:**
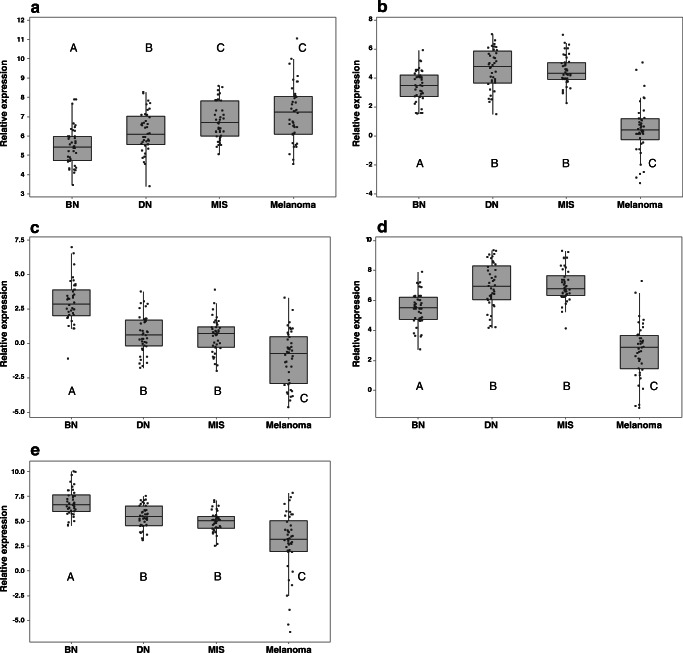
Boxplots of log-transformed relative expression of microRNAs measured with real-time PCR following correction for lesion percentage. miRNAs 21 (**a**); 200c (**b**); 204 (**c**); 205 (**d**); and 211 (**e**) in benign naevi (BN), dysplastic naevi (DN), melanoma in situ (MIS), and melanoma. Letters indicate outcome of Welch’s ANOVA followed by Games-Howell pairwise comparisons. Groups which do not share a letter are significantly different from each other (*p* < 0.05)

Following correction for lesion percentage, expression of miRNA 21 remains significantly lower in benign naevi compared to all other groups (*p* < 0.05), with expression in dysplastic naevi also significantly lower than melanoma and melanoma in situ (*p* < 0.05, Fig. 2). Expression of both miRNA 200c and 205 remains significantly lower in melanoma compared with that in all three other groups (*p* < 0.001), though following correction expression is higher in dysplastic naevi and melanoma in situ compared with that in benign naevi (*p* < 0.005). Corrected expression of miRNA 204 and 211 was significantly lower in melanoma compared with that in all other groups (*p* < 0.005) and significantly higher in benign naevi compared with that in all other groups (*p* < 0.001), with intermediate expression in dysplastic naevi and melanoma in situ. Random forest was able to correctly classify 37 out of 42 melanomas and 39 out of 42 benign naevi (Table 4). There were only two false-positive melanomas, one of which was a melanoma in situ, and only two malignant lesions were misclassified as benign naevi, one melanoma in situ, and one melanoma. The overall ROC areas remained very high for benign naevi and melanoma, at 0.98 and 0.97, respectively, and high for dysplastic naevi and melanoma in situ at 0.86 and 0.89, respectively.

**Table 4:** Confusion matrix from random forest with tenfold cross-validation following correction for lesion percentage. Each horizontal row shows how cases from each group (*BN*, benign naevi; *DN*, dysplastic naevi, *MIS*, melanoma in situ) were classified based on the miRNA expression data

	Classified as BN	Classified as DN	Classified as MIS	Classified as melanoma
BN	39	3	0	0
DN	3	27	10	1
MIS	1	13	27	1
Melanoma	1	1	3	37

### In situ hybridisation

An important consideration with the above approach to correcting for lesion percentage is that it assumes expression of all miRNAs in lesional cells only, with differences between groups reflective of differential expression in lesional cells. To further assess the spatial expression of each miRNA, in situ hybridisation with BaseScope™ was performed on a subset of cases. Due to low sensitivity of the probes resulting from the short target sequences (*direct communication with ACDbio*) and variable fixation times of clinical samples, we do not believe the results of in situ hybridisation to be accurately and reliably quantifiable. This technique has therefore only been applied to demonstrate spatial expression of miRNAs, and not as a method of quantifying miRNA expression.

**Fig. 3:**
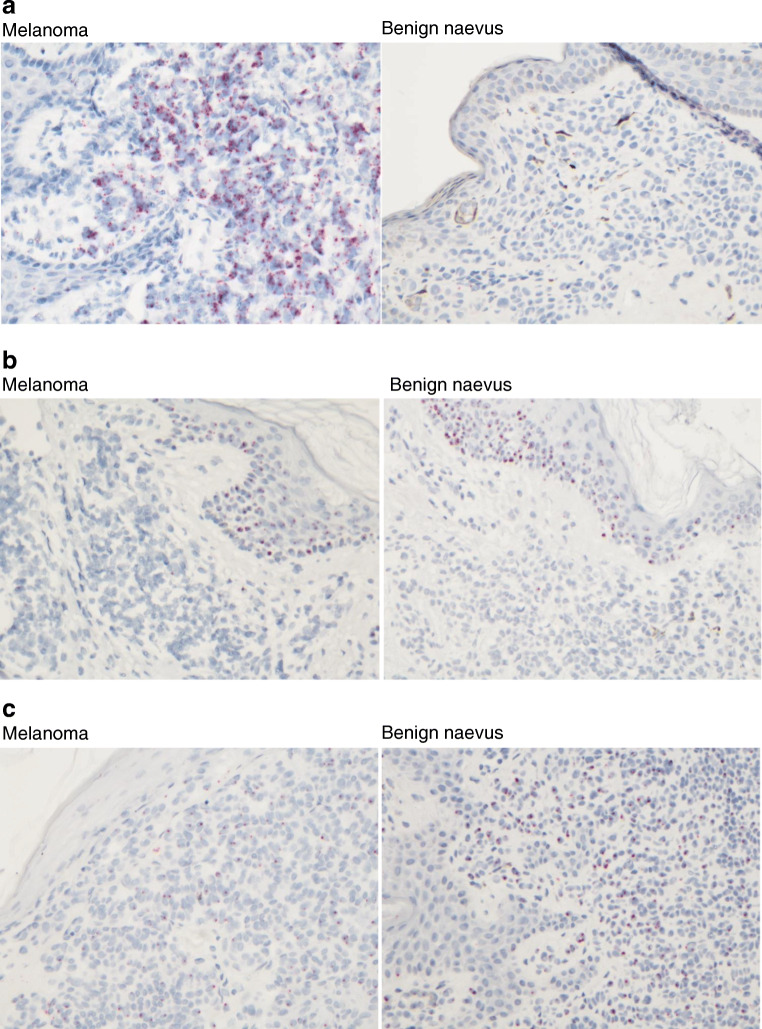
miRNA 21 (**a**), miRNA 205 (**b**), and miRNA 211 (**c**) expression in melanoma and benign naevus samples using BaseScope. **a** High expression of miRNA 21 in invasive malignant melanocytes with minimal expression in benign melanocytes, both with minimal expression in background keratinocytes. **b** Expression of miRNA 205 in keratinocytes, with minimal expression in melanocytes. **c** Expression of miRNA 211 in melanocytes, with no significant expression in keratinocytes. Black lines represent the approximate division between keratinocytes and melanocytes. All photos × 20 magnification

Despite assay optimisation, the expression of miRNA 200c and 204 remained too low to be reliably and consistently visualised. miRNA 21 was widely expressed in invasive malignant melanocytes, with minimal expression in background keratinocytes (Fig. 3a). miRNA 205 was consistently expressed in keratinocytes in both benign and malignant lesions, with minimal expression detected in benign and malignant melanocytes (Fig. 3b). miRNA 211, on the other hand, was consistently expressed in benign and malignant melanocytes, with minimal expression detected in keratinocytes (Fig. 3c). No miRNA expression was identified in other background cell types including lymphocytes and stromal cells.

## Discussion

Our study has demonstrated significantly increased expression of miRNA 21 and reduced expression of miRNAs 200c, 204, 205, and 211 in melanoma compared with that in benign naevi. This is consistent with previously published data [9, 12, 14, 15]. The expression profiles of miRNA 21 and 211 in dysplastic naevi, using a significantly larger sample size than previously published studies, are also largely consistent with previously published results [18–20]. One exception is a previous study which used fluorescent in situ hybridisation to show high miRNA 211 expression was maintained in a cohort of 21 dysplastic naevi, regardless of the degree of atypia, with significantly reduced expression in invasive melanoma [17]. We also demonstrated high sensitivity and specificity of this miRNA panel as a potential diagnostic test using a random forest machine learning algorithm.

These results, however, should be interpreted in the context of possible study limitations. It is recognised that both melanoma and melanoma in situ encompass a group of morphologically and biologically diverse lesions. We included a cohort representative of the diversity of melanoma typically encountered in diagnostic pathology practice, within which we did not find any significant differences in miRNA expression between different morphological subtypes. However, this may be appropriate for further analysis with a larger melanoma cohort including selection for rarer subtypes.

In addition, it is noted that, despite the high ROC areas resulting from the random forest algorithm, there remains a level of misclassification between dysplastic naevi and melanoma in situ which may impact clinical utility as a diagnostic test. When the miRNA expression data was scrutinised in greater detail, it was considered likely that variable lesion percentages in different groups had an impact on the final expression profiles. Correction for lesion percentage resulted in miRNAs 21, 204, and 211 showing an intermediate level of expression in dysplastic naevi and melanoma in situ, in between that of benign naevi and melanoma. Furthermore, miRNA 21 showed significantly increased levels of expression from benign naevi to dysplastic naevi to melanoma in situ, with melanoma in situ showing no significant difference from melanoma. This provides further evidence of a correlation with increasing degrees of atypia, as has been proposed previously [20]. For miRNAs 200c and 205, however, correction for lesion percentage resulted in dysplastic naevi and melanoma in situ showing a higher expression pattern than both melanoma and benign naevi, a finding which does not have any obvious biological explanation.

A further explanation for the corrected expression profiles can be sought by examining expression patterns in normal skin. Low expression of miRNA 204 and 211 in normal skin compared with that of benign naevi suggests expression only in melanocytes (which are found in low numbers in normal skin). Conversely, a similar expression of miRNA 200c and 205 in normal skin and benign naevi suggests expression in additional cell types such as keratinocytes. If there is, as suspected, expression of some of the miRNAs in background cells such as keratinocytes, this would clearly have an impact on expression levels of miRNAs measured by real-time PCR. It could also result in over-correction for lesion percentage, as some or all of the measured miRNA would be originating from background cells.

This hypothesis was tested using in situ hybridisation, which demonstrated expression of miRNA 21 and 211 principally in melanocytes, with minimal expression detected in keratinocytes. miRNA 205, however, was primarily expressed in keratinocytes, with minimal expression detected in melanocytes. Given the identical expression profiles of miRNA 200c and 205 with PCR, it is speculated that miRNA 200c may also be more highly expressed in keratinocytes than melanocytes. Similarly, given the identical expression profiles of miRNA 211 and 204, it is speculated that miRNA 204 may be expressed principally in melanocytes.

Therefore, although miRNA 205 expression is clearly associated with melanoma, the reduced expression in melanoma compared with that in all other lesion types with real-time PCR could be explained by the reduced proportion of keratinocytes in melanoma samples. Similarly, the expression in keratinocytes would account for the relatively high corrected expression in dysplastic naevi and melanoma in situ. With miRNA 21 and miRNA 211 expressed principally in melanocytes, their function as a potential biomarker of melanocytic lesions is verified. However, this also makes it difficult to be certain, when lesion percentage is not accounted for, whether low expression in dysplastic naevi and melanoma in situ with real-time PCR is due to downregulation with atypia or malignant transformation, or due to low proportion of melanocytes in these samples, or a combination of the two.

One recently published paper of particular interest examined miRNA expression in keratinocyte, melanocyte, and melanoma cell lines [15]. miRNA 204 and 211 showed significantly higher expression in cultured melanocytes than in cultured keratinocytes, while miRNA 200c and 205 showed significantly higher expression in cultured keratinocytes than in cultured melanocytes. Our study provides further support of these results by showing miRNA 211 expression principally in melanocytes and miRNA 205 expression principally in keratinocytes in human tissue samples.

The absence of significant expression of miRNA 205 in melanocytes could have considerable implications for the interpretation of the literature suggesting a role as a biomarker of melanoma, particularly as most papers examining miRNA expression profiles use techniques which result in loss of spatial expression, including miRNA microarrays, real-time PCR, and deep sequencing. Therefore, while supporting the potential for specific miRNAs including miRNA 21 and 211 to act as biomarkers of melanocytic lesions, we also highlight the crucial importance of consideration of tissue morphology and spatial expression prior to application of molecular techniques in the discovery and validation of biomarkers using human tissue specimens.

## Electronic supplementary material

ESM 1 (DOCX 6.62 MB)
