# Supplementary material for: The differential expression of micro-RNAs 21, 200c, 204, 205, and 211 in benign, dysplastic and malignant melanocytic lesions and critical evaluation of their role as diagnostic biomarkers
Source: Virchows Arch. 2020 May 9;477(1):121–30. doi: 10.1007/s00428-020-02817-5 (PMC7320036; doi:10.1007/s00428-020-02817-5)
Supplement: Supplementary file 1 — (DOCX 6.62 MB) [file 428_2020_2817_MOESM1_ESM.docx]

**SUPPLEMENTARY MATERIAL**

**ESM 1: Clinicopathological details of melanoma cohort**

| Age | Sex | Subtype of melanoma | Site | Lesion % | Ulceration | Regression | Precursor naevus | Lympho-vascular invasion | Mitosis per mm^2^ | Breslow thickness | Clark level | Microscopic satellites | Lymphocytic infiltrate | Metastasis present |
| --- | --- | --- | --- | --- | --- | --- | --- | --- | --- | --- | --- | --- | --- | --- |
| 72 | M | Superficial spreading | Leg | 75 | No | No | No | No | 12 | 2.3 | 4 | No | Absent | No |
| 79 | M | Nodular | Chest | 80 | Yes | No | No | No | 8 | 25 | 5 | No | Absent | Yes |
| 77 | F | Nodular | Arm | 80 | No | No | No | No | 6 | 7 | 4 | No | Absent | No |
| 72 | F | Superficial spreading | Knee | 75 | No | No | No | No | 12 | 3 | 4 | No | Non-brisk | No |
| 40 | F | Superficial spreading | Shoulder | 50 | Yes | Yes | No | No | 18 | 2.8 | 4 | No | Non-brisk | No |
| 41 | M | Nodular | Back | 60 | No | No | No | No | 5 | 2.8 | 4 | No | Absent | Yes |
| 85 | M | Desmoplastic and spindle cell | Shoulder | 40 | No | No | No | No | 8 | 16 | 5 | No | Non-brisk | No |
| 70 | M | Superficial spreading | Back | 65 | Yes | No | No | Yes | 15 | 23 | 5 | Yes | Absent | Yes |
| 21 | F | Nodular | Leg | 80 | Yes | No | No | No | 5 | 5 | 4 | No | Absent | No |
| 52 | F | Acral lentiginous | Foot | 70 | Yes | No | No | No | 11 | 14 | 5 | Yes | Absent | Yes |
| 90 | M | Nodular | Arm | 50 | Yes | No | No | No | 4 | 7.4 | 4 | No | Non-brisk | No |
| 72 | M | Nodular | Scalp | 70 | Yes | No | No | No | 8 | 4.2 | 5 | No | Absent | No |
| 70 | F | Superficial spreading | Arm | 80 | Yes | No | No | No | 12 | 3.8 | 4 | No | Absent | No |
| 20 | F | Nodular | Back | 50 | Yes | No | No | No | 5 | 6.5 | 5 | No | Non-brisk | Yes |
| 56 | F | Acral lentiginous melanoma | Foot | 50 | No | No | No | No | 10 | 2.5 | 4 | No | Absent | No |
| 81 | F | Nodular | Thigh | 65 | Yes | No | No | No | 4 | 2.1 | 4 | No | Absent | No |
| 68 | F | Superficial spreading | Leg | 80 | No | No | No | No | 3 | 4.2 | 5 | No | Absent | No |
| 70 | M | Desmoplastic melanoma | Scalp | 40 | No | No | No | No | 0 | 5 | 5 | No | Absent | No |
| 66 | M | Nodular | Arm | 80 | No | Yes | No | No | 8 | 1.5 | 3 | No | Absent | No |
| 69 | M | Superficial spreading | Back | 65 | No | No | No | No | 7 | 4.1 | 4 | No | Brisk | No |
| 76 | M | Desmoplastic melanoma | Scalp | 50 | No | No | No | No | 2 | 3.7 | 4 | No | Absent | No |
| 62 | M | Superficial spreading | Back | 65 | No | Yes | Yes, not dysplastic | No | 1 | 1.12 | 3 | No | Absent | No |
| 58 | M | Nodular | Scalp | 70 | No | No | No | No | 4 | 1.8 | 4 | No | Absent | No |
| 79 | F | Superficial spreading | Back | 80 | No | No | No | No | 2 | 3.4 | 4 | No | Non-brisk | No |
| 92 | M | Nodular | Back |  | No | No | No | No | 2 | 4.5 | 4 | No | Absent | No |
| 60 | M | Superficial spreading | Back | 55 | Yes | No | No | No | 21 | 4 | 4 | No | Non-brisk | No |
| 49 | M | Superficial spreading | Arm | 70 | No | Partial | No | No | 8 | 2.1 | 4 | No | Absent | Yes |
| 50 | M | Superficial spreading | Back | 50 | No | No | Yes, not dysplastic | No | 1 | 1.8 | 4 | No | Non-brisk | No |
| 57 | F | Superficial spreading | Leg | 65 | No | No | No | No | 0 | 0.9 | 4 | No | Absent | No |
| 76 | M | Nodular | Chest | 75 | No | no | no | no | 4 | 2.7 | 4 | No | Absent | No |
| 80 | F | Nodular | Face | 60 | Yes | No | No | No | 4 | 5.7 | 4 | No | Absent | No |
| 75 | M | Superficial spreading | Abdomen | 70 | Yes | Yes | No | No | 10 | 2.5 | 4 | No | Non-brisk | No |
| 40 | F | Superficial spreading | Knee | 60 | NO | No | No | Yes | 1 | 2 | 4 | No | Absent | No |
| 45 | F | Superficial spreading | Thigh | 70 | Yes | Yes | No | No | 8 | 3.2 | 4 | No | Absent | No |
| 85 | M | Nodular | Abdomen | 80 | No | No | No | No | 12 | 5.5 | 4 | No | Non-brisk | No |
| 47 | M | Superficial spreading | Leg | 70 | Yes | Yes | Yes, not dysplastic | No | 5 | 3.5 | 4 | No | Brisk | No |
| 85 | F | Nodular | Buttock | 70 | No | No | No | No | 12 | 60 | 5 | No | Absent | No |
| 64 | F | Nodular | Back | 50 | Yes | No | No | No | 8 | 5.8 | 4 | No | Non-brisk | No |
| 81 | F | Lentigo maligna | Unknown | 50 | No | No | No | No | 2 | 1.8 | 4 | No | Non-brisk | No |
| 87 | F | Superficial spreading | Shin | 50 | Yes | No | No | No | 2 | 3.5 | 4 | No | Non-brisk | Yes |
| 42 | M | Nodular | Shoulder | 72 | No | No | No | No | 16 | 2.8 | 4 | No | Non-brisk | Yes |
| 57 | F | Nodular | Back | 80 | Yes | No | No | Yes | 8 | 4.8 | 4 | No | Non-brisk | No |

A: B:

**ESM 2: Malignant (A) and benign (B) samples stained with the PPIB positive control probe using BaseScope (x20 magnification)**. Both samples demonstrate the technical performance of the assay and the integrity of RNA in the tissue samples, with consistent staining in both melanocytes and keratinocytes.

A: B:

**ESM 3: Malignant (A) and benign (B) samples stained with the DAPB negative control probe using BaseScope (x20 magnification)**.
